# Relationship Between Obesity and Depression Considering the Inflammatory Theory

**DOI:** 10.3390/ijms26114966

**Published:** 2025-05-22

**Authors:** Aleksandra Julia Oracz, Mateusz Zwierz, Maciej Naumowicz, Maria Suprunowicz, Napoleon Waszkiewicz

**Affiliations:** 1 Department of Psychiatry, Medical University of Bialystok, pl. Wołodyjowskiego 2, 15-272 Białystok, Poland; maria.suprunowicz@sd.umb.edu.pl (M.S.); napoleon.waszkiewicz@umb.edu.pl (N.W.); 2 Faculty of Medicine with the Division of Dentistry and Division of Medical Education in English, Medical University of Bialystok, Jana Kilińskiego 1, 15-089 Białystok, Poland; 39995@student.umb.edu.pl (M.Z.); 38741@student.umb.edu.pl (M.N.)

**Keywords:** obesity, adipokines, depression, inflammatory theory of depression, Th1/Th2 imbalance, metabolic biomarkers of depression

## Abstract

Numerous scientific findings indicate that excess adipose tissue, particularly visceral fat, is associated with a chronic inflammatory state manifested by elevated levels of proinflammatory cytokines and an imbalance in the T helper type 1/type 2 (Th1/Th2) response, which carries numerous metabolic consequences. Obesity induces, among other effects, the activation of the kynurenine pathway and a reduction in serotonin synthesis, alterations in adipokine profiles, modifications of the hypothalamic–pituitary–adrenal (HPA) axis, disturbances in fatty acid ratios, oxidative stress, and dysfunction of the gamma-aminobutyric acid (GABA)ergic system. These neuroimmunological and metabolic disturbances, along with obesity-induced neurotransmission abnormalities that may represent a common underlying model of depression, could provide valuable insights into the pathomechanisms of depression, allowing for prediction of disease progression and individualized therapeutic strategies in overweight patients. Furthermore, the analysis of inflammation-associated biomarkers opens up new therapeutic perspectives, suggesting that interventions aimed at reducing inflammation might lead to potential advances in the treatment of depression.

## 1. Introduction

Depression and obesity are two conditions that are correlated; however, it is essential to clearly distinguish issues of causality. Statistics unequivocally indicate that, over the years, both diseases have been diagnosed in an increasing proportion of the population—according to World Health Organization (WHO) data, the global prevalence of obesity more than doubled between 1990 and 2022 [1]. Additionally, between 2015 and 2019, a widespread increase in depression diagnoses was observed without a corresponding rise in treatment response [2]. Moreover, individuals with obesity have a 32% higher risk of developing depression compared to those with normal body weight [3].

It is important to emphasize that the etiology of depression is multifactorial. Factors include disturbances in the neurotransmission of serotonin, noradrenaline, gamma-aminobutyric acid (GABA), glutamate, and glycine, as well as theories highlighting the significant role of thyroid dysfunction [4] and abnormalities in neuronal plasticity [5]. Recently, increasing attention has been devoted to the inflammatory basis of depression, which appears especially relevant in the context of obesity, considered a state of chronic low-grade inflammation.

Furthermore, obesity is associated with low self-esteem, a distorted body image, and overall dissatisfaction with one’s appearance, which can increase vulnerability to mood disorders and intensify the tendency to develop maladaptive eating behaviors that further contribute to weight gain [6]. However, the most significant link seems to be the aforementioned association between increased adipose tissue and the resulting inflammation, which may induce or exacerbate depressive symptoms [7]. Numerous studies have demonstrated that depression is related to a chronic inflammatory state, elevated levels of T helper type 1(Th1) markers, dysregulation of the T helper type 1/T helper type 2 (Th1/Th2) ratio, and disturbances of the hypothalamic–pituitary–adrenal (HPA) axis [8].

The aim of this narrative review is to describe the relationship between excessive adipose tissue and the course of depression. An analysis of retrospective and observational studies related to the inflammatory theory of depression aims to determine the rationale for examining Th1 and Th2 response markers with respect to the relationship between immunological responses and the course of depression. Their potential value as predictors of disease progression is also evaluated, which could potentially lead to earlier medical intervention and treatment in obese patients, thereby reducing the likelihood of dangerous behaviors and improving therapeutic outcomes.

## 2. Materials and Methods

Since this work is a narrative review, no strict inclusion criteria were established for the selection of articles. Studies included were published between 2000 and 2024. The literature search was conducted on PubMed, Web of Science, and Google Scholar between October and December 2024, covering studies from 2000 to 2024 using relevant search terms, including “obesity, adipokines, depression, inflammatory theory of depression, Th1/Th2 imbalance, inflammation, and metabolic biomarkers”. Both original research and review articles were considered during the initial screening. Zotero software (version 7.0.14, 64-bit) was used to remove duplicates that appeared due to overlapping search terms. Based on titles and abstracts, conference abstracts were excluded. Studies not written in English were also omitted. This narrative review is not systematic, and despite attempts to cover all studies, one should keep in mind significant limitations.

## 3. Obesity: Causes and Link to Depression

According to the WHO definition, obesity is “abnormal or excessive fat accumulation that may impair health” [9]. It is diagnosed when the body mass index (BMI) is 30 or higher, although this measure may not accurately reflect the condition in some individuals, such as athletes [10]. Globally, it is estimated that 16% of the adult population is obese, a rate that has doubled since 1990. Among teenagers, the obesity incidence has increased four-fold [9].

**Figure 1:**
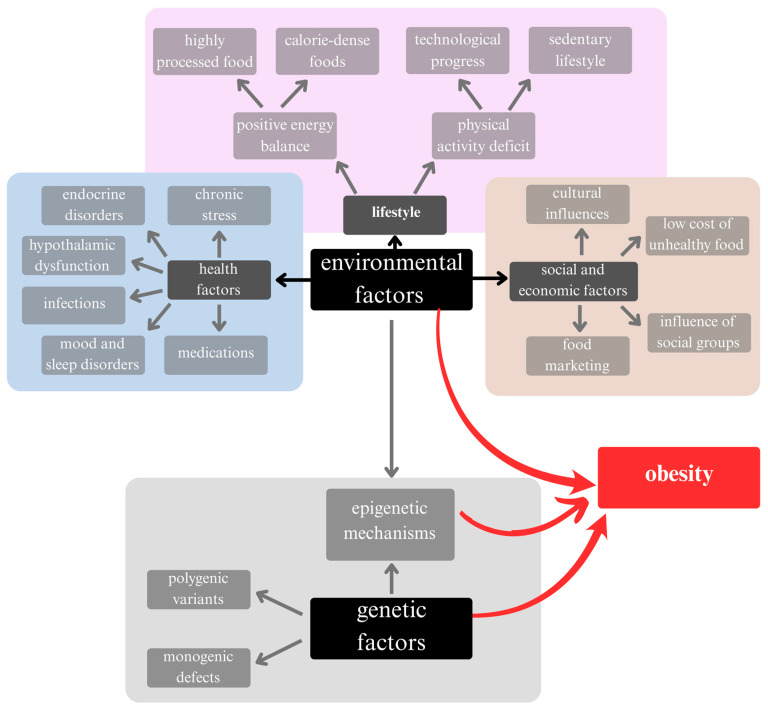
Causes of the increase in obesity in society. Genetic factors are rarely the sole cause of obesity. It has been suggested that the obesity pandemic results from the interaction of environmental and genetic factors in epigenetic mechanisms.

The development of obesity involves the interaction of genetic factors and environmental influences. While monogenic defects and polygenic variants associated with obesity have been identified, they account for only about 3% of all obesity cases [11]. It is suggested that epigenetic mechanisms, in which environmental factors influence the expression of specific genes, play a more significant role in the development of obesity. Several factors are associated with an increased incidence of obesity, including decreased physical activity (leading to lower energy expenditure), a positive energy balance (due to high-calorie, highly processed foods that are high in sugar and low in fiber), chronic stress (e.g., hormonal imbalance anad chronic inflammation), and social and living conditions (like residing in rural areas) [11,12]. Other contributing factors may include mood disorders, endocrine disorders, hypothalamic damage, and certain medications [12,13] (Figure 1).

In addition to the various complications associated with obesity, such as diabetes, heart disease, kidney disease, hypertension, and neurological disorders, obesity is also linked to an increased risk of depression [14].

## 4. Metabolic Biomarkers and the HPA Axis

Available evidence suggests that some of the aforementioned biomarkers (leptin [15,16], adiponectin [17], resistin [18], and ghrelin [19,20]) may modulate the HPA axis, which is known to be hyperactive in patients with severe depression [21,22]. Hyperactivity of the HPA axis in depression manifests as excessive secretion of corticoliberin (CRH), elevated cortisol levels, an increased cortisol response to adrenocorticotropic hormone (ACTH), impaired negative feedback mechanisms mediated by dexamethasone (DEX), and hypertrophy of the pituitary and adrenal glands [21]. This is particularly important because the HPA axis regulates the body’s response to stress, and its hyperactivity may lead to a range of depressive symptoms [16,23].

The literature emphasizes the key role of glucocorticoid secretion as a consequence of HPA axis activation, which is closely linked to the processes leading to the development of depression, as shown in studies in animal models [24], including mice subjected to chronic, unpredictable mild stress during pregnancy [25]. From the perspective of depression, the ability of these biomarkers to regulate the HPA axis is crucial, as it may help alleviate depressive symptoms associated with its hyperactivity [26]. Adiponectin, according to the hypothesis, inhibits the production of tumor necrosis factor-alpha (TNF-α), a proinflammatory cytokine that activates the HPA axis [27]. HPA axis hyperactivity has been identified as a potential contributing factor in the pathogenesis of depression [28], which may result from dysfunction of the kynurenine pathway responsible for converting tryptophan into serotonin and melatonin, compounds that regulate mood [29].

Disruption of HPA axis function due to low adiponectin levels manifests in two ways: first, as a lack of sensitivity to DEX suppression, and second, as an excessive CRH response following DEX administration [17]. The literature indicates that the administration of exogenous adiponectin produces antidepressant effects [17], suggesting that it may serve as a potential therapeutic target in the treatment of depression.

The relationship between resistin levels and HPA axis activity was investigated in a study by Weber-Hamann et al., which observed a positive correlation between serum resistin levels and free cortisol concentrations in the saliva of depressed patients [18]. This suggests that resistin may be regulated by cortisol or may influence its levels, which is supported by in vivo studies in humans [30].

The literature also indicates limited knowledge regarding the direct impact of fetuin-A (FetA) and chemerin on the HPA axis. However, their roles in neuroprotection and the modulation of inflammatory processes [31], as well as their correlation with depressive disorders [32,33], may suggest a potential indirect influence on the HPA axis. Further research is necessary to better understand these relationships.

**Figure 2:**
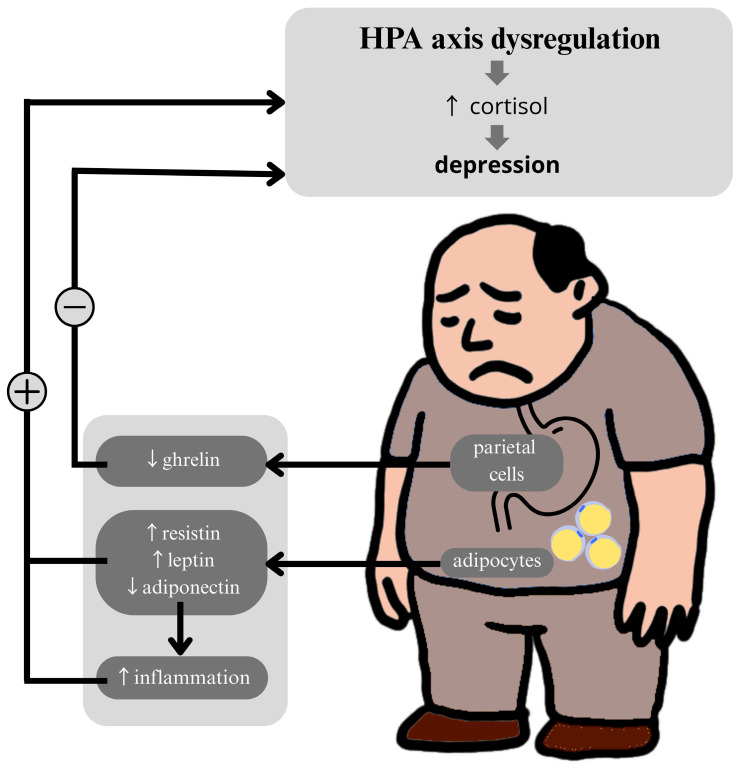
Dysregulation of the hypothalamic–pituitary–adrenal (HPA) axis in obesity. Excessive adipose tissue is responsible for increased concentrations of resistin and leptin and decreased concentrations of the anti-inflammatory adiponectin. This imbalance stimulates the HPA axis to produce cortisol, with elevated cortisol levels being linked to depression. Ghrelin secreted by the gastric parietal cells is also a hormone that stimulates the HPA axis, but its levels decrease in obesity, which may also contribute to HPA axis dysregulation: ↑ (increase), ↓ (decrease), + (stimulation), and – (inhibition).

Ghrelin also exerts a stimulatory effect on the HPA axis [20], which is mediated by the central nervous system (CNS) [19]. In the work of Van Loenen et al., it was emphasized that in stress-induced obesity, ghrelin may act in two ways: directly by stimulating ACTH in the HPA axis and indirectly by influencing hypothalamic neurons. This underscores the multifaceted impact of ghrelin on the HPA axis [20] (Figure 2).

## 5. From Obesity to Depression: The Role of the Kynurenine Pathway

Proinflammatory cytokines, whose levels are elevated in obese individuals, affect the dysfunction of the kynurenine pathway, which plays a key role in the metabolism of tryptophan (TRP). This explains why adults with obesity are more susceptible to developing depression than individuals with a normal body mass index [34,35,36]. It has been demonstrated that elevated levels of proinflammatory cytokines lead to the activation of indoleamine 2,3-dioxygenase 1 (IDO1) [37,38] and the suppression of tryptophan 2,3-dioxygenase (TDO), resulting in a shift in tryptophan metabolism [39]. Consequently, the metabolism of serotonin and melatonin is altered, leading to increased production of kynurenine (KYN) [40,41,42]. This phenomenon is consistent with the monoamine hypothesis, which postulates that depression results from a deficiency of monoamine neurotransmitters, including serotonin and noradrenaline [43]. As a result, the availability of tryptophan for serotonin synthesis is reduced, which may lead to decreased serotonin levels in the brain and, consequently, be associated with depressive symptoms [40]. Moreover, there is a strong correlation between this process and suicidal ideation and behavior, even when accounting for depression severity [44]. Such changes are particularly evident in the context of obesity, where an increased kynurenine/tryptophan ratio is observed [45], potentially contributing to an inflammatory reaction related to interferon-gamma (IFN-γ) activation [46]. Indeed, the elevated activity of proinflammatory IDO1 is closely linked with the modulation of immune and metabolic responses, as shown in studies conducted in a mouse model of induced lipopolysaccharide (LPS) and depression-like behavioral symptoms [47]. A reduction in the production of serotonin (5-HT), the main neurotransmitter, is significant for both metabolic processes and neuropsychiatric function [48]. The monoamine deficiency hypothesis suggests that a decrease in brain serotonin levels is a primary factor in the etiology of depressive disorders [49]. The ultimate consequence of the disruption of the kynurenine pathway is a reduction in the production of nicotinamide adenine dinucleotides (NAD+), which are essential for cellular energy metabolism [37]. It is noteworthy that the expression of genes encoding enzymes of the kynurenine pathway is significantly upregulated in obese individuals [44], indicating its potential adaptive or pathophysiological significance in this condition. Considering the multifaceted influence of the kynurenine pathway on immunological, metabolic [50], and neuropsychiatric processes [51], its dysfunction may serve as a potential biomarker for the development of depression in obese individuals. It is crucial to consider how inflammatory processes affect TRP metabolism, which may contribute to the onset of depressive disorders [52]. The aforementioned theories were confirmed in a study conducted by Delgado et al., which demonstrated that in more severe cases of depression, elevated levels of C-reactive protein (CRP) were associated with reduced TRP concentrations and its indole pathway metabolites, including indole-3-carboxaldehyde (IAld) [52].

**Figure 3:**
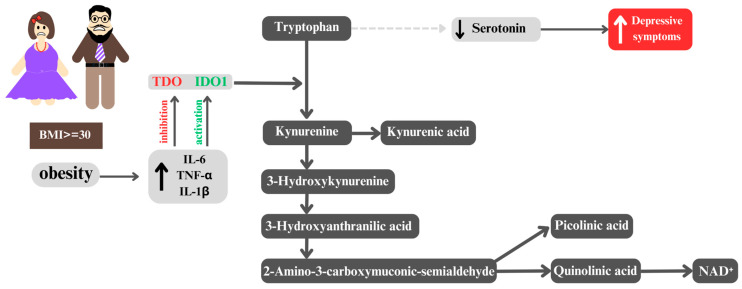
Dysregulation of the kynurenine pathway in obesity and its link to depressive symptoms. In individuals with obesity (body mass index, BMI ≥ 30), elevated levels of proinflammatory cytokines, including interleukin-6 (IL-6), tumor necrosis factor-alpha (TNF-α), and interleukin-1 beta (IL-1β), lead to the activation of indoleamine 2,3-dioxygenase 1 (IDO1) and the inhibition of tryptophan 2,3-dioxygenase (TDO). This shift in tryptophan metabolism reduces serotonin synthesis, contributing to depressive symptoms. At the same time, kynurenine is further metabolized into neurotoxic quinolinic acid, which promotes neuroinflammation, while the production of nicotinamide adenine dinucleotide (NAD+), essential for cellular energy metabolism, is altered.

It is worth noting at the outset that CRP may serve as an inflammatory marker useful in the personalized treatment of depression [53]. Large, randomized trials have shown that an increase in CRP levels above 1 mg/L is associated with a less favorable response to selective serotonin reuptake inhibitors (SSRIs) compared to catecholaminergic antidepressants [54]. Moreover, in patients who did not respond to multiple trials of conventional antidepressant treatments, elevated levels were observed not only for CRP but also for interleukin-6 (IL-6) and TNF-α [55] (Figure 3).

## 6. Metabolic Biomarkers of Depression and Obesity

Adipose tissue is a source of over 600 potentially secreted proteins that are involved in the regulation of insulin, appetite, fat distribution, energy expenditure, endothelial function, blood pressure, hemostasis, and inflammation [56].

It is well documented that excess adipose tissue, particularly visceral fat, is a source of adipokines (also known as adipocytokines) [57], which include leptin, adiponectin, chemerin, resistin, and FetA [58]. It is reasonable to assume that the presence of excess fat in the body may contribute to increased levels of inflammation, which in turn may elevate the risk of developing depression [59]. A key aspect of this process is the signaling pathway between adipocytes and the brain, mediated by adipokines [60], which significantly influences the pathophysiology of depression. This link has been demonstrated both in studies in elderly humans with late-life depression (LLD) [61] and in an animal model of chronic social-defeat stress in mice [17].

### 6.1. Leptin

Leptin, a hormone secreted by adipose tissue, is increasingly recognized as a potential biomarker for assessing depression risk, particularly in the context of obesity [57,62]. Although the primary function of leptin is the regulation of energy metabolism and appetite, it also exhibits proinflammatory effects that may contribute to increased inflammation [63]. The scientific literature reveals discrepancies regarding the relationship between leptin levels and depression [64,65,66,67]. This dual role has prompted investigations into its relevance to mental health, especially depressive disorders.

The current literature, however, presents inconsistent findings regarding the association between leptin levels and depressive symptoms. Some studies report a significant relationship between elevated leptin levels and depression [64,65], whereas others do not [66,67]. For example, one study reported no statistically significant differences in leptin concentrations between patients with depression and healthy controls [66], in contrast to other studies that found elevated leptin levels in depressed individuals, possibly reflecting symptom severity [64]. This suggests that leptin may not only be relevant in the context of obesity but may also serve as a marker for other inflammation-related processes implicated in the pathophysiology of depression [68]. Building on this framework, a number of authors have proposed that leptin resistance may provide a mechanistic link between obesity, metabolic dysfunction, and depressive symptomatology. This hypothesis is particularly relevant in the context of atypical depression, a subtype characterized by increased appetite, weight gain, and fatigue, all of which reflect disturbances in energy regulation [15,62,69].

In the study by Milaneschi et al., and in the study by Zhang et al. [70], patients with acute episodic depression exhibited a positive correlation between elevated leptin levels and symptoms characteristic of atypical depression, including increased appetite, weight gain, and muscle weakness [69]. These symptoms align with leptin’s known metabolic functions, further suggesting a pathophysiological overlap [71]. It is important to note, however, that the study by Milaneschi et al. was cross-sectional [62], thus precluding conclusions about causality. Longitudinal studies are warranted to determine whether changes in leptin levels precede, coincide with, or follow depressive symptom fluctuations over time. Notably, Zhang et al. failed to support the hypothesis that systemic inflammation mediates the relationship between leptin levels and depression [70]. The correlation between leptin levels and brain cortical areas was found to be independent of CRP levels, a common indicator of inflammation [72]. These results suggest that leptin may influence brain function through mechanisms independent of inflammatory processes [70] or through alternative inflammatory pathways that are independent of CRP, as described in the study by Burrows et al. [73].

Additional evidence indicates that elevated leptin levels, often associated with increased adiposity, may interfere with the brain’s reward-processing circuitry [73,74]. This disruption has been implicated in the development of anhedonia—defined as a diminished ability to experience pleasure—a core symptom of major depressive disorder (MDD) [75,76].

In summary, meta-analytic data underscore the heterogeneity of leptin findings in depression. A meta-analysis by Carvalho et al. revealed that these differences were not statistically significant in the general population [59]. However, a higher BMI was positively correlated with elevated leptin levels in depressed patients [59]. Patients with mild to moderate depression exhibited significantly higher leptin levels compared to controls [59], suggesting a possible compensatory role of leptin in early stages of depressive illness [77]. It has been proposed that such compensatory activity may initially counterbalance emerging metabolic dysregulation, but may become impaired over time, particularly with the progression of obesity and the onset of leptin resistance, further exacerbating both metabolic and affective disturbances, as shown in a study in mice [77].

To reconcile the discrepancies between studies, we compared original research that specifically examined the relationship between leptin levels and clinical symptoms of depression (Table 1). The heterogeneity of the findings may be due to several methodological differences. First, positive associations were more frequently observed in studies focusing on atypical depression, whereas studies reporting no significant results often did not distinguish between depressive subtypes. This suggests that the pathophysiological relevance of leptin may be subtype-specific. Second, gender and BMI appear to be critical moderators; for example, Esel et al. reported significant leptin differences only in women, and studies with samples with a higher mean BMI were more likely to detect correlations. Therefore, future studies should stratify analyses by depressive subtype, sex, and metabolic profile to clarify the role of leptin in the pathogenesis of depression.

### 6.2. Adiponectin

Adiponectin is the most abundant adipokine, secreted exclusively by mature adipocytes and primarily produced in white adipose tissue [78]. It modulates immune responses and proinflammatory cytokines, making it a potential biomarker of inflammation in depression and a differentiator between depressed and healthy individuals [57,79]. The relationship between adiponectin and depressive disorders is thought to stem from the presence of adiponectin receptors in specific brain regions involved in mood regulation, including the hippocampus [70].

Accumulating evidence indicates that individuals with MDD, including those with recurrent depressive episodes, often exhibit lower adiponectin levels [57,80]. This finding may serve as a starting point for further research aimed at improving the precision of monitoring and predicting the course of these conditions. Islam et al. suggested that adiponectin could be a valuable prognostic tool for the early assessment of depression risk [80]. However, an important limitation of these studies is the lack of consideration of potential dietary influences on the parameter being analyzed, which is significant given that adiponectin is predominantly produced by adipose tissue [81]. Future research should account for participants’ dietary habits and explore stratification by BMI and eating behaviors to refine these associations.

Although the meta-analysis by Carvalho et al. primarily addressed leptin, it also examined adiponectin and found no significant difference in adiponectin levels between depressed and non-depressed individuals across BMI categories [59]. This lack of association may reflect limitations inherent in BMI, which is a crude measure of adiposity and does not capture fat distribution [82]. More precise anthropometric assessments, such as waist circumference or body fat percentage, are recommended for future studies to clarify the adiponectin–obesity–depression axis. A recent meta-analysis by Vuong et al. supports the hypothesis that lower adiponectin levels in obese individuals may contribute to the development of inflammation and insulin resistance [83], which are recognized risk factors for depression [84]. However, this analysis did not reveal statistically significant differences in adiponectin levels between depressed and non-depressed individuals in the general population [83].

Some studies report that higher adiponectin levels are associated with lower severity of depressive symptoms [85,86], likely due to its anti-inflammatory actions [87,88]. However, findings regarding absolute adiponectin levels in depression remain inconsistent. While certain reports described reduced levels in depressed patients [80], others demonstrated elevated levels [89], or no significant differences compared to healthy controls [90]. These discrepancies may be attributed to differences in depression subtypes and severity. For example, Jeong et al. reported elevated adiponectin levels exclusively in individuals with subclinical depression, whereas no significant differences were observed in those with moderate or severe MDD compared to healthy controls [89]. In contrast, the studies by Hu et al. and Islam et al. included diagnostically heterogeneous cohorts encompassing various depressive phenotypes, which may have further contributed to the inconsistency of findings [80,90].

A study by Permody-Pachuta et al. presented intriguing data suggesting that adiponectin may be a promising biomarker for assessing the efficacy of treatment in therapy-resistant depression (TRD) [91]. The authors observed an increase in adiponectin levels in TRD patients after electroconvulsive therapy (ECT), particularly in those who showed clinical improvement [91]. This indicates a potential correlation between adiponectin and clinical recovery. In contrast, Benedetti et al. observed a positive correlation between adiponectin levels and the duration of the depressive episode, suggesting that higher hormone levels before treatment may be indicative of a poorer response to conventional pharmacotherapy [92]. Thus, adiponectin could serve as a prognostic biomarker in predicting the clinical course of depression and treatment response.

An intriguing correlation between adiponectin and chemerin was observed in a study by Malujlo-Balcerska et al., which showed decreased levels of both adipokines in patients with recurrent depressive episodes [57]. Chemerin, like adiponectin, may be an underrecognized biomarker of inflammation in depression. However, evidence for this hypothesis is limited to a few studies, including an experiment conducted on male Sprague-Dawley rats using the chronic restraint stress model [32].

### 6.3. Resistin

Resistin, another key adipokine associated with obesity [93], may play a particular role in exacerbating inflammatory states [94], suggesting a potential link between excess adipose tissue and the etiology of depression [95]. Elevated resistin levels, which correlate with higher BMI, waist circumference, and visceral fat mass [91], contribute to increased levels of proinflammatory cytokines, such as TNF-α and IL-6 [85,96].

Although the role of resistin as a mediator of inflammation in obesity is well documented [97], further research is needed to elucidate its potential direct links with depression. Available literature indicates that resistin may decrease the release of dopamine and noradrenaline in the hypothalamus, leading to reduced levels of these monoamines in synapses, a study conducted on male Wistar rats showed [98]. These effects of resistin suggest that it might increase the incidence of depressive symptoms by affecting the neurotransmission of key mood-regulating transmitters [99]. In this context, other studies have demonstrated a positive correlation between blood resistin levels and the occurrence of atypical and melancholic subtypes of major depression [18,100], which may indicate a significant role for resistin in the pathogenesis of depression. However, study results remain inconclusive. Some research reports elevated resistin levels in obese individuals [101,102], while others suggest lower levels [103]. A meta-analysis even suggested lower resistin levels in depressed patients relative to healthy controls [60]. Notably, Lehto et al. found that resistin was correlated with atypical depressive symptoms but not with typical presentations [99], and Machado-Vieira et al. reported that higher resistin concentrations were positively associated with symptom severity and decreased significantly following antidepressant treatment [85].

Further evidence highlights the potential utility of resistin as a biomarker of immune activation in mood disorders. Malujlo-Balcerska et al. proposed that elevated resistin may reflect heightened inflammatory processes and immune dysregulation in depression [68], a notion corroborated by Rahman et al., who also identified elevated serum resistin as a possible contributor to MDD pathogenesis [104]. In the context of treatment-resistant depression, a study by Permody-Pachuta et al. found that higher pre-treatment resistin levels were predictive of better clinical outcomes following ECT [91]. Of note, this study excluded overweight participants to control for the confounding effects of body weight on adipokine levels [58].

Thus far, studies on resistin have mainly focused on the correlation between overweight/obesity and its serum levels [85,97]. Parallel research continues into the potential of resistin as a marker for depression [18,99,100]. However, comprehensive studies integrating all three components—excess adipose tissue, resistin levels, and depressive symptoms—are lacking. Such analyses could provide valuable information regarding the role of resistin as a potential mediator of the mechanisms linking obesity and depression and could clarify whether a synergy between excess weight and resistin influences the risk of developing mood disorders.

### 6.4. Fetuin-A

Similar to the previously discussed resistin and leptin, elevated serum levels of FetA have been observed in patients with recurrent depressive disorder compared to controls [57]. FetA, an acute-phase glycoprotein produced by the liver and adipose tissue [104], exerts regulatory effects on inflammatory processes [31], including the inhibition of proinflammatory mediators, such as TNF-α [105]. A reduction in FetA levels may be associated with an exacerbation of inflammatory processes, as shown in studies conducted on fetuin-A-deficient mice [106].

In contrast to these findings, a study by Fanelli et al. demonstrated lower FetA lev-els in depressed patients compared to healthy controls [33]. A strength of that study was its comprehensive adjustment for potential confounders, such as age, sex, and BMI, which allowed for a more precise determination of the relationship between FetA and depressive symptoms.

Although numerous epidemiological studies indicate elevated FetA levels in obese individuals [107] and a positive correlation between FetA concentration and BMI [108], visceral adipose tissue (VAT) [109], and leptin levels [110], the number of studies analyzing the relationship between FetA and depression remains limited. As with resistin, there is a lack of comprehensive studies integrating all aspects—excess adipose tissue, FetA levels, and the severity of depressive symptoms. This gap in data represents a significant research lacuna in understanding the metabolic–inflammatory links in depression.

### 6.5. Ghrelin

Ghrelin, although not classified as an adipokine due to its primary synthesis in the gastric fundus rather than in adipocytes [111], plays a notable role in energy balance and fat accumulation, thereby indirectly linking it to obesity [112]. It promotes adiposity by reducing lipid oxidation and enhancing fat storage, ultimately contributing to weight gain [113,114].

Some studies suggest that ghrelin levels are higher in depressed individuals compared to controls [115,116], while others do not confirm such differences [117,118]. Interestingly, research by Sempach et al. examined the effect of probiotic supplementation on depressive symptoms and found that increased ghrelin levels following probiotic intake were associated with symptom improvement [119]. These findings suggest a potential mood-regulating role of ghrelin, possibly mediated by its anti-inflammatory effects. Indeed, ghrelin has been shown to suppress proinflammatory cytokines, such as IL-6 and interleukin-1 beta (IL-1β), in both in vivo models (e.g., male Sprague-Dawley rats) [120] and in vitro human T-cell cultures [121]. In contrast, ghrelin concentrations tend to be lower in obese individuals compared to those with normal BMI, a phenomenon interpreted as a compensatory adaptation to prolonged positive energy balance [122,123]. This paradoxical inverse relationship between ghrelin and body weight further complicates its role in the obesity–depression axis. Moreover, increased ghrelin levels have been observed in subgroups of depressed patients, particularly those following suicide attempts [124] and postmenopausal women with severe depression [125], suggesting that specific clinical contexts may modulate ghrelin secretion.

Animal studies have shown that ghrelin administration affects specific proinflammatory cytokines often associated with depression [126,127], leading to lower levels of these cytokines—a finding confirmed in both a clinical trial involving patients with MDD [128], as well as in a study on male Sprague-Dawley rats, in which depression was induced by a high-fat diet (HFD) and disturbed diurnal rhythm [129]. It has also been suggested that a reduction in ghrelin levels following antidepressant treatment may contribute to improved therapeutic outcomes during depressive episodes [130].

However, further studies are necessary to comprehensively analyze the relationship between obesity, depression, and ghrelin levels, as well as changes in ghrelin across different health states and its impact on inflammatory processes. To date, research on this complex interaction has been somewhat fragmented. In order to gain a fuller understanding of ghrelin’s role in modulating both inflammatory processes and depressive symptoms in the context of obesity, integrated clinical studies considering all these aspects are needed.

### 6.6. Fibroblast Growth Factor 1

Although fibroblast growth factor 1 (FGF1) has not been directly linked to the initiation of metabolic and inflammatory cascades, its potential role in the development of depressive disorders deserves attention. Peripheral administration of exogenous FGF1 exerts strong anti-diabetic effects, mediated by the FGF1 receptor (FGFR1) in adipose tissue—specifically, by lowering blood glucose levels through the inhibition of adipose tissue lipolysis [131]. It has been shown that the adipokine FGF1 enhances metabolic homeostasis in mice fed a high-fat diet, and FGF1 itself exhibits appetite-reducing effects [132].

In a study conducted by Aurbach et al., gradual administration of exogenous fibroblast growth factor 9 (FGF9) led to an increase in anxiety- and depression-like behaviors and simultaneously decreased FGFR1 expression in the dentate gyrus [133]. This may suggest that FGFR1 levels reflect both the effects of administered growth factors and the severity of depressive symptoms in animals. These findings highlight the potential of FGFR1 as a therapeutic target in the treatment of depression.

It is also worth noting that mood-stabilizing drugs, such as valproate and lithium, may regulate FGF1 gene expression [134] (Table 2).

## 7. Inflammatory Model of Depression

Available evidence supports the hypothesis that inflammation may serve as a key mechanism linking obesity with mood disorders [6]. Research on the immunological response in obesity focuses on the function of Th1 and Th2 cells, given their role in regulating inflammatory processes [135]. Th1 cells are responsible for activating macrophages, which in turn promote the cellular immune response [136]. This process is integral to the host defense mechanisms mediated by phagocytes [137]. In contrast, Th2 cells play a key role in the humoral response and are responsible for host defense mechanisms independent of phagocytes [136]. In individuals with obesity, an increase in proinflammatory cytokine levels suggests the involvement of the Th1 response [138].

Interestingly, Th1 cytokines, including IFN-γ, are elevated in the adipose tissue of obese individuals [139]. In addition, IFN-γ has been shown to facilitate the infiltration of macrophages into adipose tissue, thereby contributing to the development of a local inflammatory response, as confirmed by in vitro studies on cells of human origin [140].

The role of Th2 is to secrete cytokines, such as interleukin-4 (IL-4), interleukin-5 (IL-5), interleukin-10 (IL-10), and interleukin-13 (IL-13), which exert anti-inflammatory effects [141]. Some studies indicate that the Th2 response may have protective effects in obesity, as demonstrated in a diet-induced obesity model in mice [142]. In the early stages of obesity, a simultaneous activation of both Th1 (proinflammatory) and Th2 (anti-inflammatory) responses is observed. The presence of mixed Th1 and Th2 cytokines in the serum of individuals with metabolic syndrome [143,144] indicates the involvement of both the innate and adaptive immune systems [145]. However, as the disease progresses, the levels of both types of cytokines decline, which may indicate a weakening of immune function in advanced obesity [142]. This phenomenon supports the concept that obesity should be regarded as a state of chronic, low-grade inflammation [146]. Such an inflammatory state is characterized by activation of the innate immune system, accumulation of proinflammatory macrophages in adipocytes [147], and elevated levels of acute-phase proteins and cytokines, such as CRP, TNF-α, IL-1β, and IL-6 [135], which are indicators of a Th1 response [148]. The inflammatory and cytokine model of depression suggests that an imbalance in the immune system—such as increased levels of proinflammatory cytokines or decreased production of anti-inflammatory cytokines—may be a key factor in the pathogenesis of clinical depression [149,150,151].

A substantial body of evidence supports the correlation between pathological behaviors—such as anhedonia, social withdrawal, and reduced activity—and elevated levels of proinflammatory cytokines, particularly TNF-α and IL-6 [149,152,153]. These findings were confirmed in a 2009 meta-analysis by Dowlati et al., which reported significantly elevated TNF-α and IL-6 levels in individuals with depressive disorders compared to healthy controls [153]. Interestingly, no significant differences were found for IL-1β or IFN-γ in the same analysis [153], nor for anti-inflammatory cytokines, such as IL-4 and IL-10.

A more recent study by Amerikanou et al. investigated the relationship between inflammatory markers (IL-6 and TNF-α) and mental well-being in obese individuals. Notably, the correlation between TNF-α and depression severity (measured via the Center for Epidemiologic Studies Depression Scale) remained statistically significant only in males [6], which contrasts with previous findings suggesting greater susceptibility of females to inflammation-related psychiatric conditions [154]. This discrepancy may stem from the relatively small sample size in the study, potentially limiting generalizability. Man et al. observed similar cytokine patterns in patients with bone tumors and depression—IL-6 and IL-1β levels were elevated and correlated with Hamilton Depression Rating Scale (HAMD-17) scores, while anti-inflammatory cytokines, such as IL-10 and transforming growth factor beta 1 (TGF-β1), were decreased prior to treatment. After sertraline therapy, proinflammatory cytokines decreased and anti-inflammatory cytokines increased, suggesting a modulatory effect of antidepressants on immune status [155]. However, the lack of a comparison group receiving sertraline without depression remains a limitation.

A meta-analysis conducted by Lombardi et al. confirmed that IL-6 levels tend to be elevated in depressed patients compared to healthy individuals, underscoring the role of inflammation in the pathogenesis of depression [156]. Various therapeutic strategies, including pharmacotherapy, have also demonstrated reductions in IL-6, further supporting its involvement in symptom modulation [156,157,158]. Moreover, proinflammatory cytokines, such as IL-6 and TNF-α, can cross the blood–brain barrier [159], influencing neurotransmission (e.g., GABAergic signaling) and contributing to mood dysregulation [160]. 

A meta-analysis by Islam et al. revealed that TNF-α levels were elevated in depressed patients compared to healthy subjects [161]. In addition, no significant differences were found in the levels of CRP and IFN-γ between the groups [161]. A potential limitation of this meta-analysis is that it focused on cytokines in peripheral fluids rather than in the CNS, where key processes associated with depression occur. Including CNS cytokines could provide a more comprehensive picture of the issue. Another meta-analysis of 13 studies involving 1123 patients examined the relationship between CRP levels and depressive symptom severity [162]. The analysis revealed a significant correlation between elevated CRP levels and depressive symptom severity, with 10 out of 13 studies demonstrating a positive correlation [162]. This meta-analysis confirmed the correlation, indicating a moderate effect size.

**Figure 4:**
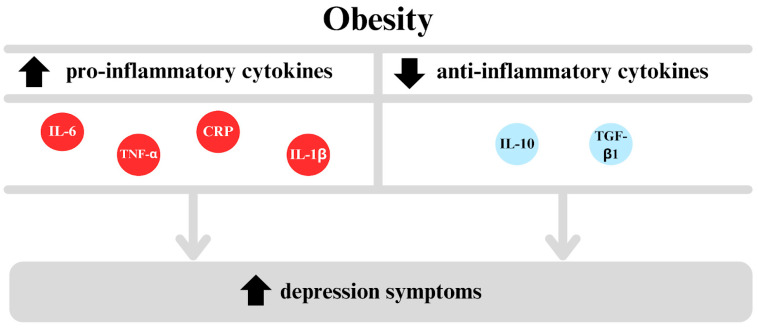
The inflammatory model posits a link between obesity and depression, proposing that obesity leads to an increase in proinflammatory cytokines, including IL-6, TNF-α, C-reactive protein (CRP), and interleukin-1 beta (IL-1β), while concurrently decreasing levels of anti-inflammatory cytokines, such as interleukin-10 (IL-10) and transforming growth factor beta 1 (TGF-β1). This immune imbalance has been linked to an elevated risk of depression symptoms, thereby supporting the hypothesis that chronic low-grade inflammation may play a role in the development of mood disorders.

In light of the above studies, it can be stated that among the discussed proinflammatory cytokines, IL-6, CRP, and TNF-α are the most promising candidates as potential biomarkers for depression. This is not only because they have been the subject of the most extensive research but also because their elevated levels have been demonstrated in many of the analyses described above. Moreover, their significance is particularly noteworthy in the context of obesity, where the levels of these cytokines are also elevated [135]. Therefore, IL-6, CRP, and TNF-α may represent promising targets in research on the pathomechanisms of depression and in the search for new diagnostic strategies (Figure 4).

## 8. Oxidative Stress

An increasing body of evidence indicates that oxidative stress (OS), defined as the overproduction and accumulation of free radicals, plays a key role in the pathogenesis of depression [163]. Analysis of keywords in scientific publications shows that “oxidative stress” has become one of the most important terms in recent years, suggesting growing interest in this issue in the context of depression [164]. At the same time, obesity—a state of chronic, low-grade inflammation [146]—is significantly correlated with elevated levels of oxidative stress markers [165].

**Figure 5:**
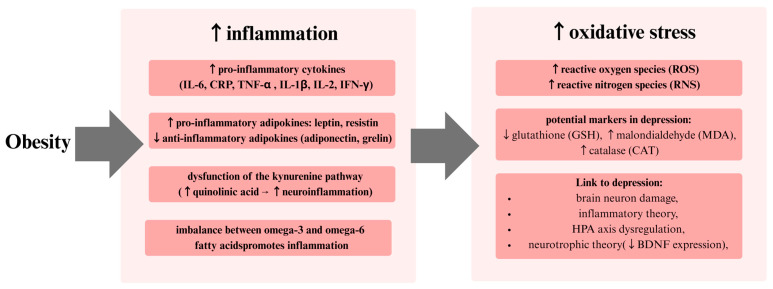
Excessive adipose tissue in obesity promotes inflammation through various mechanisms. Inflammation, in turn, exacerbates oxidative stress, which is associated with depression. Abbreviations used in the figure: interferon-γ (IFN-γ), brain-derived neurotrophic factor (BDNF).

Oxidative stress is defined as a pathological condition in which the production of free radicals, such as reactive oxygen species (ROS) and reactive nitrogen species (RNS), exceeds the organism’s capacity to neutralize them [166,167]. The literature indicates that the brain is particularly susceptible to oxidative stress due to its high oxidative metabolism, the presence of polyunsaturated fatty acids in cell membranes, and the limited activity of enzymatic systems responsible for neutralizing ROS [168,169,170,171]. Oxidative stress has been linked with other theories of depression, including HPA axis dysregulation [172,173], the inflammatory theory (where excessive synthesis of proinflammatory cytokines is associated with reduced antioxidant activity, and neuroinflammation induces excessive ROS production) [174,175,176], and the neurotrophic theory (where overproduction of ROS inhibits the expression of BDNF) [175]. The observed correlation between obesity, oxidative stress, and depression suggests a complex mechanism in which excess adipose tissue leads to increased oxidative stress, which in turn may contribute to a higher risk of depression [177]. In line with this, a study by Sanaeifara et al. demonstrated that oxidative stress plays a central role in the link between type 2 diabetes (T2D) and depression, further underscoring the involvement of obesity—a major risk factor for T2D—in this process [177,178]. Scientific literature emphasizes the particular significance of enzymes, such as superoxide dismutase (SOD) and glutathione (GSH), in assessing oxidative stress in patients with depression, serving as markers of oxidative stress in depressive disorders [174,179,180]. A study by Eshkevari et al. found that individuals diagnosed with MDD had lower glutathione levels compared to healthy subjects, suggesting that GSH could serve as a potential biomarker for depression and that its supplementation may provide therapeutic benefits [181]. A study by Duffy et al., investigating the effects of omega-3 fatty acid supplementation on GSH levels in brain tissues of elderly individuals at risk for depression, produced interesting results [182]. These fatty acids possess anti-inflammatory and antioxidant properties [183]. However, supplementation did not lead to the expected increase in GSH levels, and the observed increase in the placebo group was unexpectedly correlated with a worsening of depressive symptoms, as measured by the Patient Health Questionnaire-9 (PHQ-9) [183]. Currently, there are few comprehensive studies that simultaneously analyze BMI as an indicator of obesity, GSH levels, and their relationship with depression. However, intriguing reports in the literature indicate a significant inverse correlation between serum GSH levels and BMI [184]. In a separate study by Foulds et al., examining the relationship between SOD levels and depression, it was observed that serum SOD levels might be decreased, while SOD levels in erythrocytes might be increased [185]. In contrast, Stefanescu et al. reported reduced serum SOD activity [186], whereas Camkurt et al. found decreased SOD activity in erythrocytes [187]. The scientific literature on the relationship between SOD activity and depression is inconclusive—some studies indicated increased SOD activity in depressed patients [188,189,190], while others observed a decrease [186,191] or no differences compared to healthy individuals [192,193]. Nevertheless, the latest meta-analysis did not demonstrate a clear trend in SOD activity in patients with MDD [194]. The potential of malondialdehyde (MDA), a product of lipid peroxidation, as a biomarker of oxidative stress in depression is not widely recognized in the literature [195]. A study by Islam et al. found that serum MDA levels were significantly elevated in individuals diagnosed with MDD compared to controls [196]. These results have been echoed by other studies on lipid peroxidation in MDD and its modulation by antidepressant therapy, which have shown that MDA levels tend to decrease following effective treatment [197,198,199]. The significance of MDA as a potential biomarker of depression is further reinforced by its correlation with body mass, as studies have shown that MDA levels are significantly higher in obese individuals compared to those with normal BMI [200]. These observations suggest a potential link between obesity and increased oxidative stress, emphasizing the need for further studies on the relationship between MDA and obesity [201]. Despite the abundance of studies highlighting the importance of oxidative stress biomarkers—including catalase (CAT), glutathione reductase (GR), and glutathione S-transferase (GST)—in the etiology of bipolar disorders [202], only a few articles address potential associations between CAT and depression [194]. Moreover, there is a paucity of literature examining the correlation between depression and GR or GST. Some studies reported increased CAT activity in depressed patients, a finding supported by meta-analyses [189,190,194,203]. With regard to GR and GST, there is a lack of data. In contrast, findings concerning glutathione peroxidase (GPX) are more varied—some studies indicate decreased GPX in depressed patients [186,204], others report increased levels [184], while additional studies have found no statistically significant differences [189,192] (Figure 5).

When discussing the issue of oxidative stress, it is essential to highlight the role o nuclear factor erythroid 2-related factor 2 (NRF2). In response to oxidative stress, the Keap1–Cul3 complex becomes inactivated, leading to the stabilization and activation of NRF2. The activated NRF2 translocates to the cell nucleus, where it binds to the antioxidant response element (ARE), initiating the expression of genes encoding antioxidant and detoxifying enzymes [205]. Moreover, NRF2 contributes to the suppression of inflammatory responses by inhibiting NF-κB pathway activation and limiting the production of proinflammatory cytokines [206]. Given its pivotal role in inflammation, NRF2 may also influence the development of depressive disorders through its anti-inflammatory mechanisms [207]. This is supported by rodent studies showing that mice exposed to chronic stress exhibit depression-like symptoms [208] along with reduced levels of Keap1 and NRF2 proteins in the CA3 region of the hippocampus, dentate gyrus, and prefrontal cortex compared to healthy controls [209].

The existing literature on oxidative stress parameters, obesity, and depression is inconclusive and often contradictory. There is a clear gap in the literature providing consistent data that definitively elucidates the mechanisms linking these factors, highlighting the need for further research. A better understanding of the role of oxidative stress in these interactions could contribute to a more nuanced comprehension of depression’s pathogenesis and the development of novel diagnostic and therapeutic strategies.

## 9. Fatty Acids and Cholesterol

A significant correlation has been identified between obesity and depression, with lipid biomarkers—such as polyunsaturated fatty acids (PUFAs) and cholesterol fractions (HDL—high-density lipoprotein, LDL—low-density lipoprotein, and VLDL—very low-density lipoprotein)—emerging as key factors in the genesis of this phenomenon [210]. These biomarkers not only serve structural functions in the body but also play a role in regulating inflammatory processes [211,212]. PUFAs, particularly omega-3 and omega-6 fatty acids, act as precursors to eicosanoids, compounds with potent anti-inflammatory or proinflammatory actions [213]. An imbalance in the omega-3 to omega-6 ratio—a phenomenon frequently observed in individuals with obesity—may exacerbate inflammatory processes, thereby affecting brain function and increasing the risk of depression [214]. The anti-inflammatory action of omega-3 fatty acids largely depends on their incorporation into cell membranes, where they compete with arachidonic acid (AA) for binding sites [215]. AA is a precursor to many proinflammatory mediators, including prostaglandins and leukotrienes [216]. Replacing AA with omega-3 fatty acids, such as eicosapentaenoic acid (EPA) and docosahexaenoic acid (DHA), has been shown to reduce the production of proinflammatory eicosanoids [215] and increase the synthesis of anti-inflammatory compounds, including resolvins and protectins [217]. Multiple studies have shown that individuals with depression exhibit reduced levels of EPA and DHA in erythrocyte membranes, which may play a key role in the pathogenesis and severity of depressive symptoms [218,219]. An observational study found a correlation between national omega-3 fatty acid intake and depression prevalence, indicating that populations with higher consumption levels tend to report lower rates of depressive disorders [220]. Additionally, supplementation with omega-3 fatty acids may be particularly effective in patients with severe depressive episodes, with EPA appearing more effective than DHA in therapeutic outcomes [221]. However, some studies have yielded inconclusive results. In a study by Bidzan-Wiącek et al., although supplementation led to a marked increase in EPA and DHA blood levels—compounds with known anti-inflammatory and neuroprotective effects [222]—it did not affect depressive symptoms. This outcome may be explained by the absence of diagnosed depression among study participants [223].

In addition to examining the function of omega-3 fatty acids, a review article emphasized the importance of omega-6 fatty acids in the context of mental health [172]. A high n-6/n-3 ratio may trigger systemic inflammation, whereas a lower ratio—achieved through higher omega-3 intake—appears to offer protective effects. Therefore, maintaining a low n-6/n-3 dietary ratio is considered a key component in both prevention and treatment of depression [172]. Conversely, a study focusing on omega-6 fatty acid metabolism found no significant differences in omega-6 levels between individuals with a history of mood disorders and healthy controls, suggesting that omega-6 fatty acids may not be a major contributor to depression pathogenesis [224]. 

Cholesterol, in addition to its role in cell membrane structure [225] and steroid hormone synthesis [226], plays a key role in neuronal function, including myelin synthesis and the modulation of membrane receptors [227]. Alterations in cholesterol metabolism, including changes in the concentrations of its various fractions, are increasingly recognized as significant factors associated with the occurrence of depression [228,229]. Furthermore, elevated levels of LDL cholesterol and total cholesterol, along with reduced HDL cholesterol levels, are frequently observed in individuals with obesity [230]. Such metabolic abnormalities may be significant in the context of mental health, as cholesterol has a proven impact on the integrity of neuronal cell membranes, as demonstrated in in vitro studies conducted primarily on primary cultures of Sprague-Dawley strain rat hippocampus neurons and HEK-293 cell lines [231]. Some studies indicate that low serum cholesterol may be associated with suicidal ideation [212], while others have reported elevated cholesterol levels in individuals with major depression [232]. These inconsistent findings reflect the complexity and controversy in the current literature. For instance, while some reports associate low cholesterol with depression [233], others emphasize the limitations of such studies due to reliance on self-report questionnaires rather than clinical diagnosis. 

A notable U-shaped relationship between LDL cholesterol levels and depression risk was observed in a U.S. population study: both very low and very high LDL levels were linked to a higher incidence of depression in men, while in women, low HDL levels—but not LDL—were associated with depression [234]. A study by Wagner et al. found significantly elevated LDL cholesterol levels in patients during a major depressive episode (MDE), with higher baseline levels predicting better treatment outcomes, suggesting LDL may serve as a biomarker for both depression severity and response to therapy [235]. A meta-analysis by Persons and Fiedorowicz supported the relationship between cholesterol and depression, showing lower serum LDL cholesterol levels in individuals with depression, which is consistent with Engelberg’s hypothesis that reduced neuronal membrane cholesterol disrupts serotonin receptor function, impairing neurotransmission [229,236]. Additionally, elevated non-HDL cholesterol has also been correlated with a higher depression risk [237].

In a more detailed analysis, Wysokiński et al. discussed issues related to cholesterol fractions, observing that patients with unipolar depression exhibited higher HDL cholesterol levels and lower LDL cholesterol levels compared to individuals with schizophrenia and bipolar affective disorder [228]. However, it should be noted that this study was limited by the absence of a healthy control group. Including such a control group could help clarify the relationship between cholesterol fractions and psychiatric disorders, thereby enabling the identification of potential biomarkers specific to unipolar depression. Research into the role of triglycerides as potential biomarkers in psychiatric disorders, including depression, is still in its early stages. One of the few studies addressing this issue, conducted by Laederach-Hofmann et al., observed that depressed patients may exhibit elevated triglyceride levels [173].

## 10. GABAergic System

Dysfunction of the GABAergic system is increasingly recognized as a potential factor linking obesity with psychiatric disorders, including depression, as shown in a study in mice [238]. An expanding body of research indicates that in individuals with obesity, GABA—the primary inhibitory neurotransmitter in the CNS [239]—can modulate inflammatory processes in adipose tissue, as confirmed in both mouse studies and adipose tissue samples from obese individuals [240], as well as in a mouse model of obesity induced by a HFD [241]. This modulation impacts both metabolic processes and nervous system function [242]. Moreover, GABA is implicated in the pathogenesis of obesity by influencing metabolic disturbances and excessive food intake [243]. An animal study using a HFD-induced obesity model showed that the liver in obese individuals produces and releases increased amounts of GABA into the bloodstream, leading to inhibition of vagus nerve activity and promoting the development of hyperinsulinemia and insulin resistance [243]. The anti-inflammatory potential of GABA in adipose tissue has been highlighted by studies demonstrating its ability to reduce macrophage infiltration and proinflammatory cytokine levels (including TNF-α and IL-6), thereby improving insulin sensitivity in diet-induced obese mice [240,244]. However, these effects were observed only in subcutaneous adipose tissue (IAT) and not in visceral adipose tissue (EAT), which, as shown in a study in mice, may indicate a selective sensitivity of IAT to GABA [240]. Nevertheless, other experimental studies, also conducted on mice with HFD-induced obesity, suggest a broader role for GABA in the pathophysiology of obesity. GABA has been shown to reduce visceral fat accumulation while promoting the growth of muscle mass, which may prevent the development of both abdominal and sarcopenic obesity [244]. Furthermore, additional research indicates that GABA exerts anti-obesity effects by inhibiting fat accumulation, increasing energy expenditure, and improving metabolic parameters, as demonstrated in a mouse model of HFD-induced obesity and in vitro studies in adipocyte line 3T3-L1 [245]. GABA plays a critical role in maintaining neurochemical balance, and its dysfunction is increasingly linked to psychiatric disorders, including depression [246]. The potential link between obesity and depression may lie in common inflammatory mechanisms [129,146]. Obesity is defined as a state of chronic low-grade inflammation that affects both adipose tissue [146] and the central nervous system [129]. Proinflammatory cytokines, such as IL-6 and TNF-α, can cross the blood–brain barrier [159], thereby affecting neurotransmission—including GABAergic pathways—which may contribute to the development of mood disorders [160]. A large body of literature supports the notion that depressed patients exhibit lower GABA levels in cerebrospinal fluid [247,248]. These findings were confirmed in a meta-analysis by Romeo et al. involving 26 studies, which demonstrated significantly lower GABA levels in both serum and cerebrospinal fluid in patients experiencing an active episode of major depression compared to controls [249]. A pivotal study by Godfrey et al., utilizing magnetic resonance spectroscopy (MRS), showed a decrease in GABA levels in brain regions associated with depressive disorders [250]. Notably, this study also found that GABA levels returned to normal once the depression subsided [250], suggesting a potential correlation between GABA dysfunction, the pathogenesis of the disorder, and the treatment process. These results are in line with earlier studies reporting similar changes in GABA concentrations in the brains of depressed patients [251,252]. A positron emission tomography (PET) study using the [¹¹C]-flumazenil tracer revealed reduced binding of the GABA-A receptor in patients with depressive disorders in specific brain regions, including the hippocampal gyrus and the right superior temporal cortex [253]. These receptor deficits may be directly related to depressive symptoms, particularly those associated with impaired emotional processing and cognitive function mediated by these brain regions [254]. Other studies have also identified a significant involvement of GABA-A receptors in the mechanisms of depression [255]; however, not all findings are consistent. For example, a similar study by Persson et al. did not demonstrate differences in GABA-A receptor binding between depressed patients and healthy controls [256]. These discrepancies may result from methodological differences or the heterogeneity of depression itself, underscoring the need for further research. Additional evidence for GABA system dysfunction in depression comes from postmortem studies showing reduced expression of glutamate decarboxylase 67 (GAD67), a key enzyme involved in GABA synthesis, in the prefrontal cortex of depressed individuals [257,258]. However, not all studies agree. Some analyses did not find differences in GAD67 expression in the prefrontal cortex between psychiatric patients, including those with depression, and controls [259,260]. These discrepancies may be due to differences in study design, including whether patients were receiving antidepressant treatment or were drug naïve. The hypothesis that GABA system dysfunction is a causative factor in depression has been supported by the efficacy of drugs acting as positive allosteric modulators of GABA-A receptors [261], which have shown beneficial effects in the treatment of depression [262]. Nevertheless, GABA dysfunction is not exclusively associated with depression [263]. Moreover, reduced levels of GABA and related biomarkers have been documented in other psychiatric disorders, including schizophrenia and bipolar affective disorder [264,265]. These findings suggest that GABA system impairment may be a more general factor in the development of psychiatric disorders rather than a specific marker for depression.

## 11. Limitations

Due to the review nature of the work, systematic inclusion and exclusion criteria were not applied, nor was a critical appraisal of the methodology used in the studies performed. The work includes both small- and large-group studies that are characterized by heterogeneous data. Discrepancies in the results of the cited studies impede a clear interpretation of the role of certain biomarkers in the pathogenesis of depression in individuals with obesity.

## 12. Summary

An increasing body of scientific evidence indicates that the chronic inflammatory state induced by excess adipose tissue plays a key role in the pathogenesis of psychiatric disorders. The inflammatory and cytokine model of depression suggests that an imbalance in the immune system—characterized by elevated proinflammatory cytokine levels or reduced production of anti-inflammatory cytokines—may be a critical factor in the development of clinical depression. Among the proinflammatory cytokines discussed, IL-6, CRP, and TNF-α appear to be the most promising candidates as potential biomarkers for depression, particularly in the context of obesity, where their levels are also elevated.

Furthermore, excess adipose tissue, especially visceral fat, is associated with increased production of adipokines, which may exacerbate inflammatory processes. Within this group, leptin, which exerts proinflammatory effects, shows inconsistent associations with depression, with some studies reporting elevated levels in depressed individuals and others finding no significant differences. Similarly, adiponectin, which has anti-inflammatory properties and is often lower in obese individuals, may reflect a weakening of protective immune mechanisms, while resistin, which enhances inflammatory processes and modulates neurotransmission, has been identified as a potential biomarker to differentiate depressed individuals from healthy subjects, though study results remain variable. FetA also demonstrates variable findings in relation to depression, indicating a need for further investigation. Interesting observations have also been made regarding ghrelin, an energy-regulating hormone that can influence inflammatory processes and depressive symptoms. Additionally, disturbances in the kynurenine pathway—where activation of IDO1 reduces the availability of tryptophan for serotonin synthesis—may contribute to depression. Another key aspect addressed is the impact of metabolic biomarkers on the HPA axis, whose hyperactivity is associated with depressive symptoms. An imbalance in the omega-3 to omega-6 ratio exacerbates inflammation, thereby increasing the risk of depression. Furthermore, increased oxidative stress—measured through glutathione levels and antioxidant enzyme activities—and GABAergic system dysfunction play crucial roles in the mechanisms linking obesity with mood disorders.

The literature presented suggests that excess adipose tissue may initiate a cascade of metabolic and inflammatory disturbances that indirectly elevate the risk of depression. Discrepancies among study findings may be attributed to differences in methodology, characteristics of the populations studied, or the measures used to assess obesity. Rather than relying solely on BMI, which does not fully capture the distribution of adipose tissue, assessing body fat percentage or waist circumference may be more appropriate. Biomarker analysis provides valuable insights into the underlying mechanisms of these interactions, allowing for prediction of the course of psychiatric disorders and the personalization of therapeutic strategies in patients with excess body weight. The conclusions open new therapeutic perspectives, suggesting that interventions aimed at reducing inflammation may be beneficial in the treatment of depression. A comprehensive approach that considers both metabolic and immunological aspects appears to be crucial for the effective diagnosis and treatment of these disorders.

This review provides a significant extension of the existing literature by not only synthesizing current findings on the inflammatory basis of depression in the context of obesity but also drawing attention to underexplored biomarkers, such as FetA and chemerin. Unlike the widely studied proinflammatory cytokines (IL-6, TNF-α, and CRP), these markers are rarely investigated in psychiatric research, despite their potential as indicators of both the metabolic and inflammatory background of depression. This identified gap highlights the need for studies integrating adipokine levels with mental health status, metabolic parameters, and neuroimaging data.

**Figure 6:**
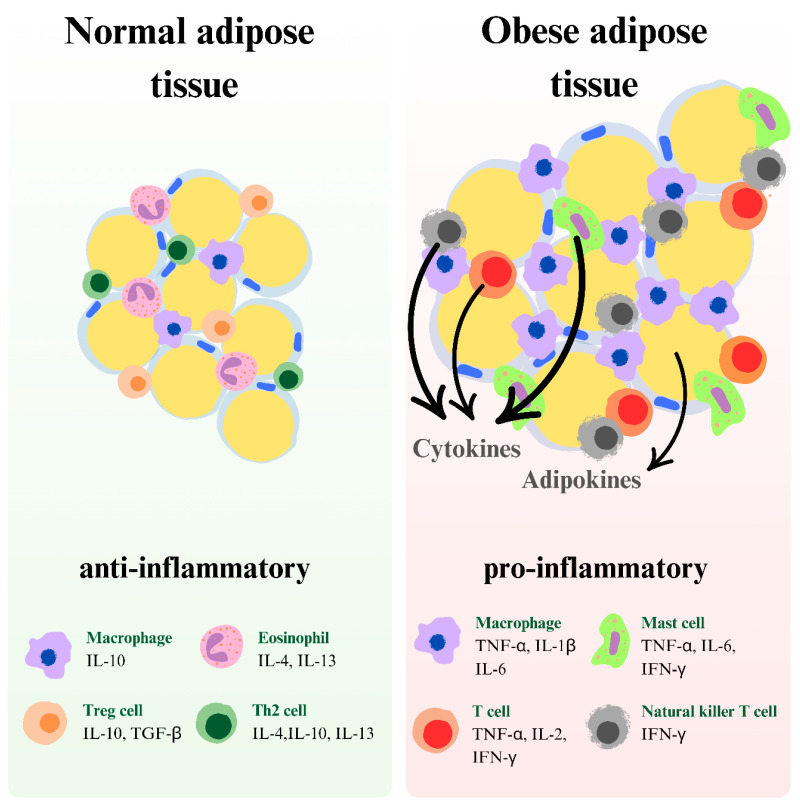
The graphic depicts the variations in immune cell composition and cytokine patterns between normal and obese adipose tissue. Normal adipose tissue is characterized by an anti-inflammatory environment, with macrophages producing IL-10, eosinophils releasing interleukin-4 (IL-4), IL-10, and interleukin-13 (IL-13), regulatory T cells (Treg) secreting IL-10 and transforming growth factor beta (TGF-β), and Th2 cells producing IL-4, IL-10, and IL-13. In contrast, obese adipose tissue exhibits a proinflammatory profile with macrophages secreting TNF-α, IL-1β, and IL-6, mast cells producing TNF-α, IL-6, and IFN-γ, T cells releasing TNF-α, interleukin-2 (IL-2), and IFN-γ, and natural killer T cells producing IFN-γ.

Furthermore, the article proposes an integrative pathophysiological model that connects immunological mechanisms, metabolic factors, hormonal regulation, neurotransmitter alterations, and oxidative stress into a unified pathway linking obesity and depression. This approach emphasizes the multifactorial nature of the interaction between these two conditions and supports the need for holistic diagnostics and treatment strategies that target not only depressive symptoms but also their metabolic and inflammatory underpinnings (Figure 6).

## Figures and Tables

**Table 1:** Summary of studies investigating the relationship between BMI, leptin levels, and depression symptom severity.

Authors and Year	BMI Differences	Depressive Symptom Severity and Subtype	Key Findings
Milaneschi et al., 2017 [62]	• Current MDD group: mean BMI = 25.8 ± 5.3 kg/m^2^. • CG: mean BMI = 25.1 ± 4.5 kg/m^2^.	• Three MDD classes identified based on IDS-SR30: severe typical, severe atypical, and moderate. • Melancholic and atypical subtypes were examined.	• Higher leptin levels were associated with the atypical subtype of MDD. • No association found between leptin and overall MDD diagnosis or melancholic subtype.
Burrows et al. (2024) [64]	• MDD group: mean BMI = 30.41 ± 4.61 kg/m^2^. • CG: mean BMI = 25.1 ± 4.5 kg/m²	• Patients with MDD showed moderate to severe depressive symptoms (assessed using PROMIS Depression Score). • The study did not specify MDD subtypes (e.g., melancholic or atypical).	• Higher serum leptin levels were observed in the MDD group compared to CG.
Esel et al. (2005) [65]	• MDD group: mean BMI = 25.68 ± 4.59 kg/m^2^ (♂ 23.92 ± 3.58; ♀ 26.74 ± 4.88). • CG: mean BMI = 24.49 ± 3.28 kg/m^2^ (♂ 23.43 ± 3.33; ♀ 26.13 ± 2.56)	• Patients with MDD showed moderate to severe depressive symptoms (assessed using MADRS). • The study did not specify MDD subtypes (e.g., melancholic or atypical).	• Women with MDD had significantly higher leptin levels than healthy women in the CG. • No difference in leptin levels was found between men with MDD and healthy men.
Heinen et al. (2023) [66]	• MDD group: mean BMI = 24.7 ± 0.4 kg/m^2^. • CG: mean BMI = 24.6 ± 0.7 kg/m^2^.	• Patients with MDD showed moderate depressive symptoms (assessed using BDI-2). • The study did not specify MDD subtypes (e.g., melancholic or atypical).	• No significant differences in leptin levels were found between MDD patients and healthy controls.
Sohan et al. (2023) [67]	• MDD group: mean BMI = 23.57 ± 0.308 kg/m^2^. • CG: mean BMI = 24.45 ± 0.26 kg/m^2^.	• Patients with MDD showed moderate depressive symptoms (assessed using HAM-D). • The study did not specify MDD subtypes (e.g., melancholic or atypical).	• No significant differences in serum leptin levels were observed between MDD patients and healthy controls.
Zhang et al. (2024) [70]	• MDD group: mean BMI = 28.69 ± 5.47 kg/m^2^ (sample I); 27.16 ± 5.59 kg/m^2^ (sample II). • CG: BMI = 27.90 ± 5.66 kg/m^2^ (sample I); 24.86 ± 4.76 kg/m^2^ (sample II).	• Patients in both samples showed at least moderate, and likely severe, depressive symptoms (assessed using PHQ-9 and PROMIS Depression Score). • The study specifically focused on atypical depressive symptoms, defined as sleep problems, fatigue, and appetite changes.	• Higher leptin levels were correlated with increased severity of atypical depressive symptoms.

The table includes only original research studies referenced in Section 6.1, comparing findings on leptin levels and depressive symptom severity. Abbreviations used in the table: major depressive disorder (MDD); control group (CG); Inventory of Depressive Symptomatology Self-Report (30 items) (IDS-SR30); Patient-Reported Outcomes Measurement Information System Depression Score (PROMIS Depression Score); Montgomery–Åsberg Depression Rating Scale (MADRS); Beck Depression Inventory-II (BDI-2); Hamilton Depression Rating Scale (HAM-D); Patient Health Questionnaire-9 (PHQ-9); ♂ (male); ♀ (female).

**Table 2:** A comparative summary of key metabolic markers, their mechanisms of action, and their roles in obesity and depression. The table categorizes adipokines as either proinflammatory or anti-inflammatory and outlines their altered levels in obesity and depression.

Metabolic Marker	Mechanism of Action	Marker Level in Obesity	Effect in Obesity	Marker Level in Depression
Leptin	proinflammatory	↑ [57,62]	↑ inflammation	↑ [64,65,70], no significant difference [67]
Adiponectin	anti-inflammatory	↓ [83]	↑ inflammation	↓ [57,80,83,86], no significant difference [59,90], ↑ [87]
Resistin	proinflammatory	↑ [91]	↑ inflammation	↑ [18,99,100,101,102,104], ↓ [60,103]
Ghrelin	anti-inflammatory	↓ [122,123]	↑ inflammation	↑ [115,116], no significant difference [117,118]
Fetuin-A	anti-inflammatory	↑ [108]	↓ inflammation	↑ [57], ↓ [33]

Fibroblast growth factor 1 (FGF1) was not included in the table due to the fact that it has not been directly linked to the initiation of metabolic and inflammatory cascades.
